# Distribution of FFRCT in single obstructive coronary stenosis and predictors for major adverse cardiac events: a propensity score matching study

**DOI:** 10.1186/s12880-022-00783-9

**Published:** 2022-03-31

**Authors:** Xianglan Jin, Xiangyu Jin, Xiaoyun Wu, Luguang Chen, Tiegong Wang, Wangfu Zang

**Affiliations:** 1 Department of Cardiac Surgery, Shanghai Tenth People’s Hospital, Tongji University School of Medicine, No. 301 Yanchang Middle Road, Shanghai, 200072 China; 2 Hainan College of Economics and Business, Haikou, 571127 Hainan China; 3 Department of Radiology, Changhai Hospital, Naval Medical University, No. 168 Changhai Road, Shanghai, 200433 China

**Keywords:** Coronary artery disease, Fractional flow reserve, Tomography, X-ray computed, Prognosis

## Abstract

**Background:**

Fractional flow reserve derived from computed tomography (FFRCT) has been demonstrated to improve identification of lesion-specific ischemia significantly compared with coronary computed tomography angiography (CCTA). It remains unclear whether the distribution of FFRCT values in obstructive stenosis between patients who received percutaneous coronary intervention (PCI) or not in routine clinical practice, as well as its association with clinical outcome. This study aims to reveal the distribution of FFRCT value in patients with single obstructive coronary artery stenosis and explored the independent factors for predicting major adverse cardiac events (MACE).

**Methods:**

This was a retrospective study of adults with non-ST-segment elevation acute coronary syndrome undergoing FFRCT assessment by using CCTA data from January 1, 2016 to December 31, 2020. Propensity score matching (PSM) method was used to account for patient selection bias. The risk factors for predicting MACE were evaluated by a Cox proportional hazards regression analysis.

**Results:**

Overall, 655 patients with single obstructive (≥ 50%) stenosis shown on CCTA were enrolled and divided into PCI group (279 cases) and conservative group (376 cases) according to treatment strategy. The PSM cohort analysis demonstrated that the difference in history of unstable angina, Canadian Cardiovascular Society Class (CCSC) and FFRCT between PCI group (188 cases) and conservative group (315 cases) was statistically significant, with all *P* values < 0.05, while the median follow-up time between them was not statistically significant (24 months vs. 22.5 months, *P* = 0.912). The incidence of MACE in PCI group and conservative group were 14.9% (28/188) and 23.5% (74/315) respectively, *P* = 0.020. Multivariate analysis of Cox proportional hazards regression revealed that history of unstable angina (adjusted odds ratio (adjOR), 3.165; 95% confidence interval (CI), 2.087–4.800; *P* < 0.001), FFRCT ≤ 0.8 (OR, 1.632;95% CI 1.095–2.431; *P* = 0.016), and PCI therapy (OR 0.481; 95% CI 0.305–0.758) were the independent factors for MACE.

**Conclusions:**

History of unstable angina and FFRCT value of ≤ 0.8 were the independent risk factors for MACE, while PCI therapy was the independent protective factor for MACE.

## Background

Fractional flow reserve (FFR) is an invasive technique for assessment of flow limitation in patients with coronary artery disease (CAD) [1]. An FFR value of ≤ 0.8 is generally considered to best identify flow obstruction and the strongest predictor of improved clinical outcome after coronary revascularization [2]. The FFR for guiding percutaneous coronary intervention (PCI) trials have demonstrated that patient with obstructive CAD can benefit from coronary revascularization [3, 4]. FFR derived from computed tomography (FFRCT) is a novel technique for assessing the physiological significance of CAD [5]. FFRCT has been demonstrated to improve identification of lesion-specific ischemia significantly compared with coronary computed tomography angiography (CCTA) stenosis grading alone [6].

It remains unclear whether the distribution of FFRCT values in obstructive coronary artery stenosis between patients who received PCI or not in routine clinical practice, as well as its association with clinical outcome. Therefore, the purpose of this study was to reveal the distribution of FFRCT value in patients with single obstructive coronary artery stenosis and explored the independent factors for predicting major adverse cardiac events (MACE), by using a propensity score matching (PSM) method to balance the covariates between those who received PCI therapy or not. We hypothesized that history of unstable angina and FFRCT value of ≤ 0.8 were the independent risk factors for MACE, while PCI therapy was the independent protective factor for MACE.

## Methods

### Study population

This retrospective study was approved by the Ethics Committee of our Hospital (IRB protocol number: 2020-KN11-01) and written informed consent was waived. Adult patients with non-ST-segment elevation acute coronary syndrome (NSTE-ACS) (aged > 18 years) and underwent coronary computed tomography angiography (CCTA) in our hospital, between January 1, 2016 and December 31, 2020, were identified. Patients with single obstructive (≥ 50%) stenosis shown on CCTA were enrolled. Exclusion included patients with ST-segment elevation myocardial infarction (STEMI), PCI that performed in multi-vessels, prior coronary artery bypass grafting (CABG), history of malignant tumor. Subsequently, patients were divided into conservative group and PCI group according to treatment strategy.

### CCTA acquisition

All patients underwent CCTA on a 320 row CT-scanner (Aquilion One Vision, Toshiba Medical Systems, Tokyo, Japan) with a collimation of 320 × 0.5 mm and a tube rotation time of 0.5 s. Tube current was set between 200 and 550 mAs at 120 kV, adjusting primarily mAs according to body habitus. Axial scanning was performed with prospective ECG-gating at 30–80% of the R-R interval, with a section thickness of 0.75 mm. A bolus of 50 ml iobitridol (Xenetix-350; Guerbet, France) was intravenously injected at a speed of 5 ml/s, followed by a 20 ml saline flush. A SureStart technique was used to trigger the scan, with a region of interest placed in the descending thoracic aorta with a threshold of 300 Hounsfield Units (HU).

### Image analysis

Two senior radiologists (L.C., 10 years experience and T.W., 12 years experience) independently interpreted the CCTA data using a workstation (Vitrea fX 6, Vital Images), by consensus to visually assess for percent luminal stenosis of left anterior descending (LAD), left circumflex (LCX), and right coronary (RCA). A single coronary artery with ≥ 50% stenosis was considered obstructive CAD.

Evaluation of FFRCT was performed by an operator (BLINDED, 10 years experience in cardiac CT) using a dedicated software (uCT-FFR version 1.5, United Imaging Healthcare, Shanghai, China). FFRCT values were derived from CCTA data as following steps: (1) anatomic model reconstruction; (2) centerline definition; (3) boundary condition; and (4) uCT-FFR calculation [7]. Another radiologist (BLINDED, 15 years experience in CT post-processing) reviewed the post-processed images to verify the value of FFRCT was properly calculated. Any lesion vessel indicating a FFRCT value of ≤ 0.8 was defined as ischemic lesion [8].

### Clinical outcome

All enrolled patients were followed up by two cardiologists via telephone or outpatient clinic visit. The primary outcome was a MACE, which was defined as all-cause mortality, hospitalization for MI, cardioembolic stroke, hospitalization for unstable angina, or urgent coronary revascularization [9]. All outcomes were ascertained up to May 31, 2021.

### Statistical analysis

Statistical analysis was conducted using IBM SPSS software (Version 19.0, IBM Statistical Package for the Social Sciences, Chicago, IL). Quantitative variables were represented as the means ± standard deviations (SD) or the medians (interquartile ranges: IQRs), and categorical variables were expressed as absolute frequencies and proportions. Clinical characteristics and imaging parameters were compared between PCI group and conservative group using the Mann–Whitney U test or chi-squared test when appropriate.

PSM method was used to account for patient selection bias. Patients in PCI group and conservative group were then matched 1:4 using nearest neighbor matching with a caliper of 0.2 times the SD of the propensity scores. A Cox proportional hazards regression analysis was used to investigate the factors for predicting MACE in the matched sample. Factors associated with MACE in unadjusted Cox models (*P* < 0.2) in the matched sample were included in the multivariable models to identify the independently risk factors associated with MACE. All results were presented as odds ratios (ORs) and 95% confidence intervals (CIs). A two-tailed *P* value < 0.05 was considered statistically significant.

## Results

### Clinical characteristics

**Fig. 1:**
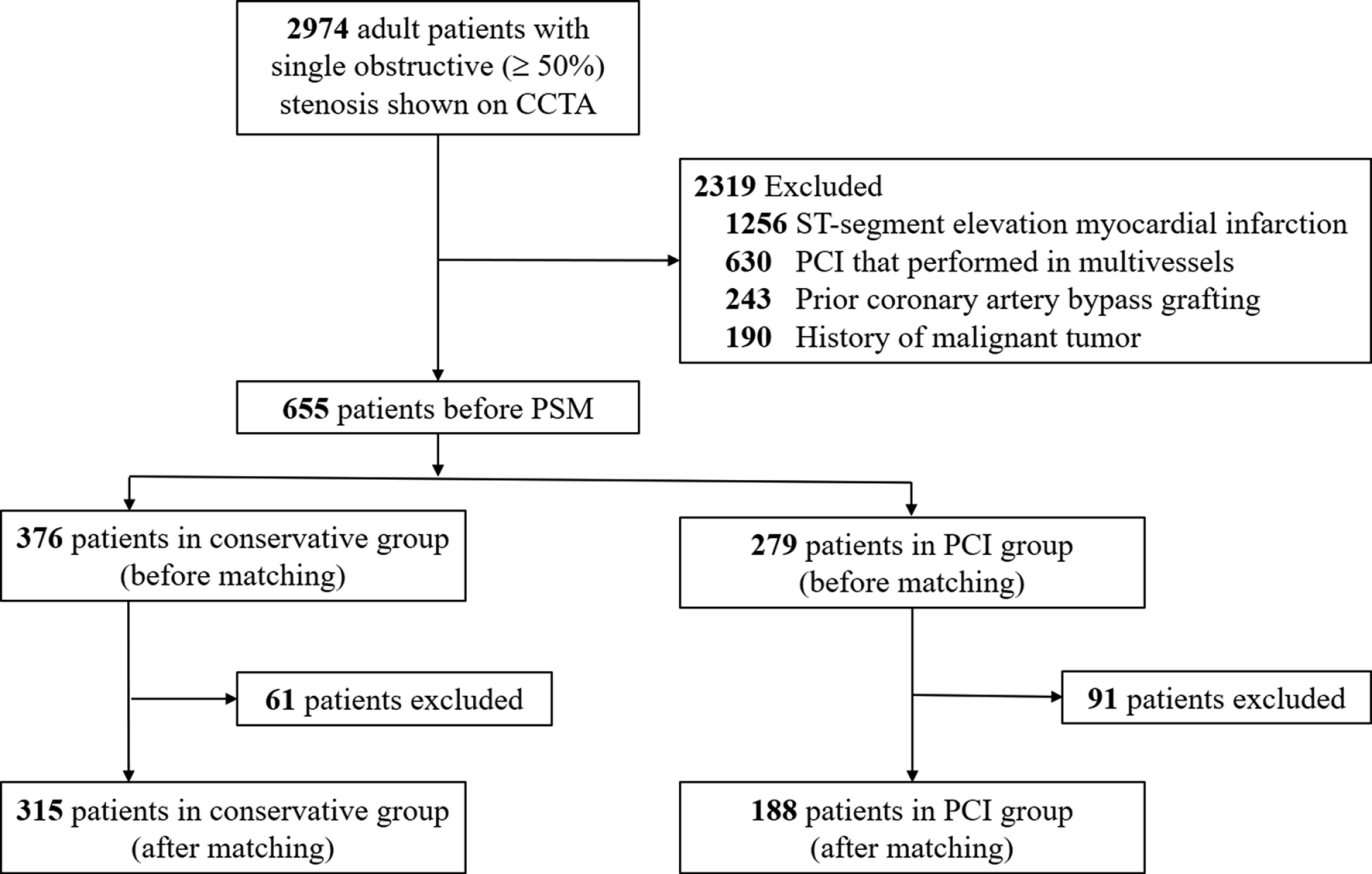
Flowchart of the study. CCTA, coronary computed tomography angiography; PCI, percutaneous coronary intervention; PSM, propensity score matching

Overall, there were 655 unique patients (376 in conservative group and 279 in PCI group) with single obstructive stenosis during the study period. After PSM analysis, 503 matched patients (315 in conservative group and 188 in PCI group) were finally included for further analysis (Fig. 1). An overview of clinical characteristics before and after PSM is presented in Table 1. PSM cohort analysis demonstrated that only the difference in history of unstable angina, Canadian Cardiovascular Society Class (CCSC) and FFRCT value between the two groups were statistically significant, with all *P* values < 0.05.

**Table 1:** Baseline characteristics before and after propensity score matching

Baseline characteristics	Baseline characteristic		*P* value	Baseline characteristics		*P* value
	Before PSM			After PSM		
	Conservative group (n = 376)	PCI group (n = 279)		Conservative group (n = 315)	PCI group (n = 188)	
Male, n(%)	254(67.6)	202(72.4)	0.182	221(70.2)	131(69.7)	0.910
Age, Mean (SD), years	66.8 ± 9.5	64.6 ± 10.2	0.005	66.5 ± 9.7	65.3 ± 10.3	0.195
≤ 65 n(%)	172(45.7)	149(53.4)	0.052	152(48.3)	90(47.9)	0.934
> 65 n(%)	204(54.3)	130(46.6)		163(51.7)	98(52.1)	
Smoking, n(%)	85(22.6)	101(36.2)	< 0.001	80(25.4)	57(30.3)	0.230
Hypertension, n(%)	192(51.1)	178(63.8)	0.001	178(56.5)	108(57.4)	0.837
Diabetes, n(%)	96(25.5)	67(24.0)	0.657	80(25.4)	51(27.1)	0.669
Hyperlipidemia, n(%)	19(5.1)	13(4.7)	0.817	16(5.1)	8(4.3)	0.675
Unstable angina, n(%)	111(29.5)	190(68.1)	< 0.001	111(35.2)	103(54.8)	< 0.001
CCSC, n(%)						
0	38(10.1)	0(0)	< 0.001	24(7.6)	0(0)	< 0.001
1	183(48.7)	121(43.4)		150(47.6)	86(45.7)	
2	117(31.1)	141(50.5)		106(33.7)	88(46.8)	
3	38(10.1)	17(6.1)		35(11.1)	14(7.4)	
FFRCT, median (IQR)	0.85(0.70, 0.91)	0.75(0.61, 0.86)	< 0.001	0.83(0.69, 0.89)	0.77(0.65, 0.87)	0.008
≤ 0.8, n(%)	147(39.1)	173(62.0)	< 0.001	140(44.4)	108(57.4)	
> 0.8, n(%)	229(60.9)	106(38.0)		175(55.6)	80(42.6)	0.005
MACE, n(%)	77(20.5)	41(14.7)	0.035	74(23.5)	28(14.9)	0.020

CCSC, Canadian cardiovascular society class; IQRs, interquartile ranges; MACE, major adverse cardiac events; PCI, percutaneous coronary intervention; PSM, propensity score matching; SD, standard deviation

### Clinical outcomes

In the matched sample, the median follow-up time was 22.5 (12, 32) months in conservative group and 24 (12, 27) months, the difference between them was not statistically significant, with *P* = 0.912. Finally, the overall incidence of MACE was 20.3% (102/503), of whom 2 patients died from sudden cardiac death, 3 patients suffered STMI, 6 patients had a cardioembolic stroke, 91 patients were re-hospitalized due to unstable angina (34 cases) or urgent coronary revascularization (57 cases). The incidence of MACE in PCI group was significantly reduced than conservative group (14.9% vs 23.5% respectively), with a P value of 0.020.

### Multivariate Cox proportional hazards regression analysis for MACE

**Fig. 2:**
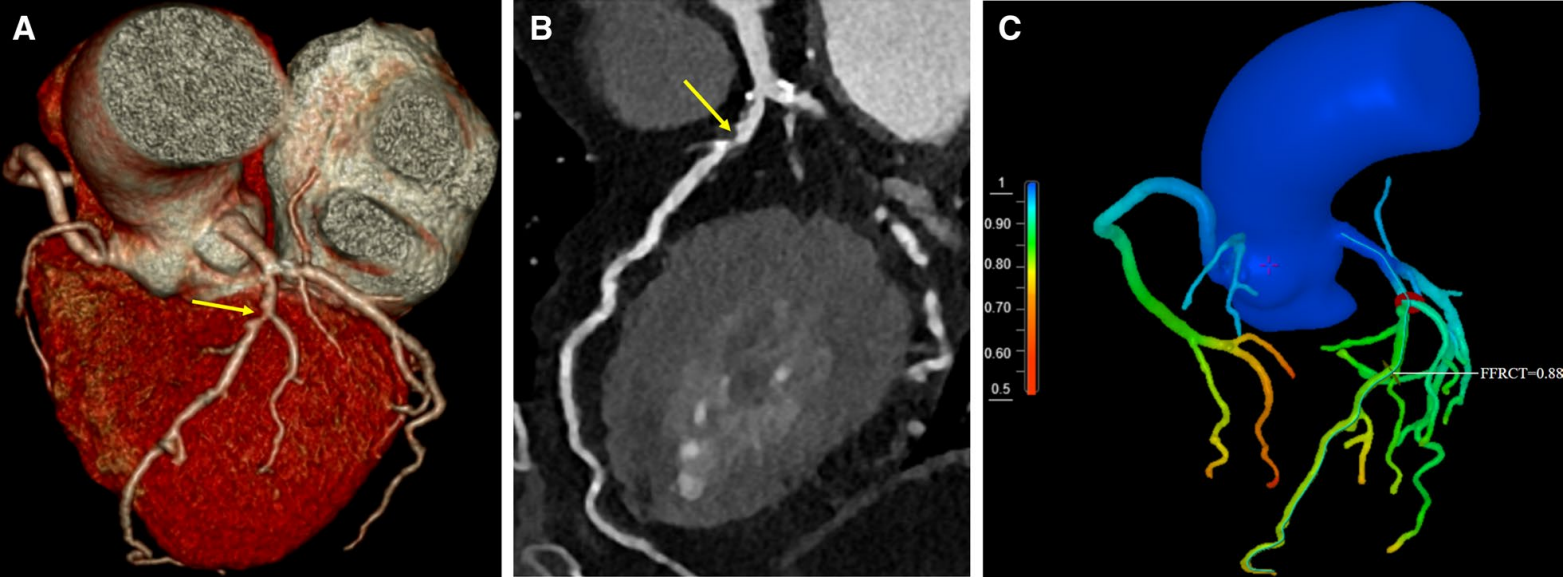
Representative case of patient without MACE. A 78-year-oid female with unstable angina. CCTA showed mixed plaque with moderate stenosis at proximal LAD (yellow arrow in **A**, **B**). This lesion was revealed to be hemodynamically insignificant with FFRCT value of 0.88 (**C**). The patient was treated with medical therapy and was followed up for 24 months without MACE. CCTA, coronary computed tomography angiography; FFRCT, fractional flow reserve derived from computed tomography; MACE, major adverse cardiac events; LAD, left anterior descending

**Fig. 3:**
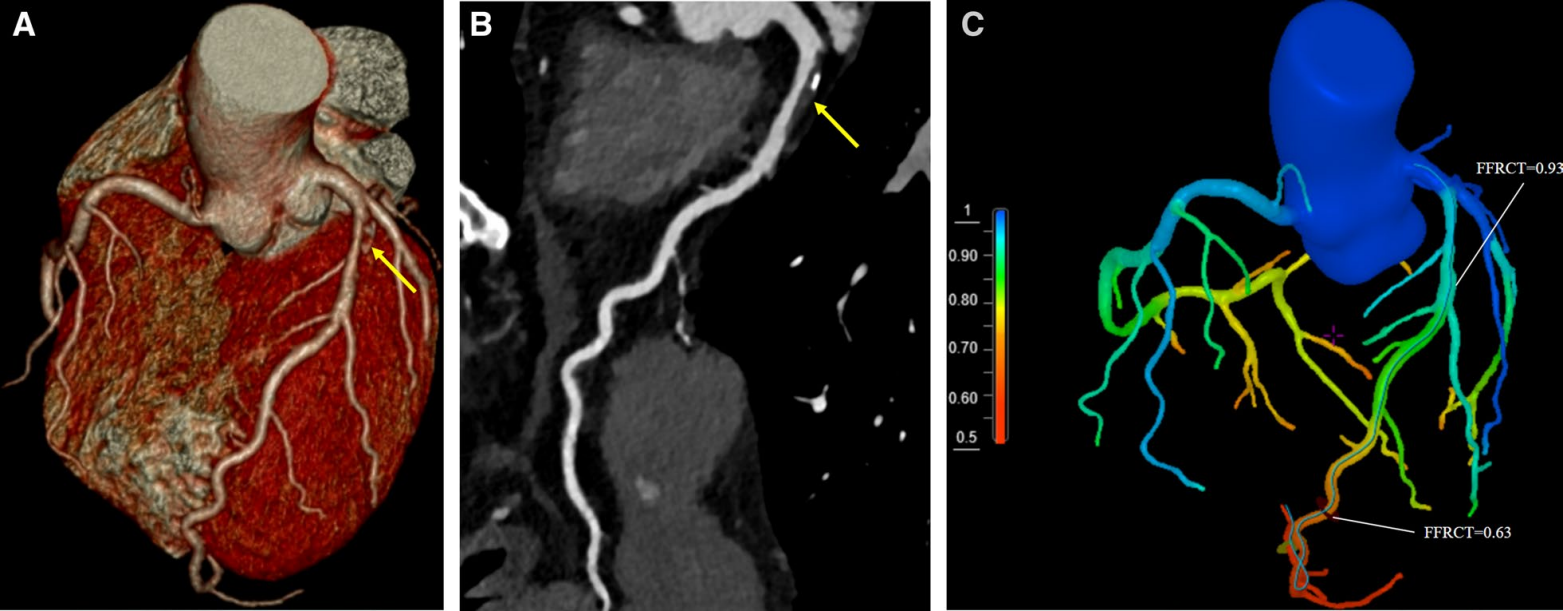
Representative case of patient with MACE. A 58-year-oid male with unstable angina. CCTA showed mixed plaque with moderate stenosis at proximal LAD (yellow arrow in **A**, **B**). This lesion was revealed to be hemodynamically insignificant with FFRCT value of 0.93 at proximal LAD, while hemodynamically significant with FFRCT value of 0.63 at distal LAD (**C**). The patient was treated with medical therapy initially, but 2 months later he suffered a sudden acute anterior myocardial infarction, and underwent emergency revascularization. CCTA, coronary computed tomography angiography; FFRCT, fractional flow reserve derived from computed tomography; MACE, major adverse cardiac events; LAD, left anterior descending

After adjusted by multivariate Cox proportional hazards regression model, it revealed that history of unstable angina (adjusted odds ratio (OR) 3.165; 95% confidence interval (CI) 2.087–4.800; *P* < 0.001), FFRCT ≤ 0.8 (OR 1.632; 95% CI 1.095–2.431; *P* = 0.016) were the independent risk factors for MACE, while PCI therapy (OR 0.481; 95% CI 0.305–0.758) was the independent protective factor for MACE (Table 2). Representative cases without and with MACE are shown in Figs. 2 and 3.

**Table 2:** Cox proportional hazards regression analysis after PSM (n = 503)

Variables	Univariate analysis			Multivariate analysis		
	OR	95% CI	*P* value	OR	95%CI	*P* value
Male	1.242	0.796–1.940	0.339			
Age > 65, years	1.135	0.768–1.677	0.525			
Smoking	1.184	0.776–1.808	0.434			
Hypertension	0.742	0.502–1.097	0.133			
Diabetes	1.148	0.743–1.774	0.535			
Hyperlipidemia	1.103	0.449–1.713	0.831			
Unstable angina	2.638	1.761–3.952	< 0.001	3.165	2.087–4.800	< 0.001
CCSC						
1	2.735	0.656	− 11.408	0.167		
2	4.248	1.025	− 17.615	0.046		
3	5.058	1.140	− 22.432	0.033		
FFRCT ≤ 0.8	0.155	0.042–0.572	0.005	1.632	1.095–2.431	0.016
PCI therapy	0.717	0.464–1.110	0.136	0.481	0.305–0.758	0.002

CCSC, Canadian cardiovascular society class; CI, confidence interval; OR, odds ratios; PCI, percutaneous coronary intervention; PSM, propensity score matching

## Discussion

This study revealed the distribution of FFRCT value in patients with single obstructive coronary artery stenosis, and explored the independent factors for predicting MACE, by using a PSM method to balance the covariates between those who received PCI therapy or not. It showed that PCI group had lower FFRCT values than conservative group, and history of unstable angina, ischemic lesion, and PCI therapy were independent factors for MACE.

In this study, approximately 44.4% of patients with conservative therapy who had ischemic lesions did not undergo PCI, which was higher than previous studies [10, 11]. The reasons might include the absence of symptoms, old age, patient preferences [12]. In present study, only 35.2% of patients had unstable angina, besides 55.2% had a grading of CCSC lower than 2, showing that most of patients in conservative group had a good symptom status. There were relatively more patients with ischemic lesions in PCI group than that in conservative group (57.4% vs. 44.4%) in this matched sample. Moreover, PCI therapy was associated with lower incidence of MACE during the follow-up in patients with obstructive coronary artery stenosis. In other words, patients could benefit from PCI therapy in the current study, which was consistent with the result of Sud et al. [10]. On the contrary, the recent ISCHEMIA trial demonstrated that there was not significant difference in MACE between the conservative and invasive strategy [13]. A major difference is in ISCHEMIA they enrolled patients with stable coronary disease, meanwhile the current work included patients with NSTE-ACS.

The frequency of PCI for nonischemic lesions (FFRCT > 0.8) observed in the present study, 38.0% before PSM and 42.6% after PSM shown in Table 1, was much higher than that reported previously [12, 14]. This may be due to that the PCI strategy in this retrospective study were mostly depending on assessment of luminal stenosis rather than guidance of invasive FFR or non-invasive FFRCT. Besides, clinicians may opt to perform PCI for nonischemic lesions to prevent from MACE in patients with unstable angina or FFRCT is not routinely calculated in CCTA. PCI therapy was considered significantly associated with better clinical outcomes in ischemic lesions and worse outcomes in nonischemic lesions, compared with conservative strategy [10]. The DEFER clinical trial with extended follow-up at 15 years indicated that a higher risk of late MI was observed after PCI for nonischemic lesions [15, 16].With the wide application of non-invasive FFRCT, patients with nonischemic lesions would receive appropriate treatment options.

In our study, FFRCT value of ≤ 0.8 was considered ischemic lesion, which was independent risk factor for predicting MACE. However, this was not in line with that reported by Yu et al. [17], myocardial blood flow rather than FFRCT was the strongest predictor for MACE in that study. This difference may be ascribed to the reduced diagnostic accuracy of FFRCT in the presence of severe vessel calcification [18, 19]. In addition, myocardial blood flow represented the myocardial perfusion status, while FFRCT only suggested the hemodynamic significance of epicardial vessel stenosis.

The analysis of non-invasive FFRCT in this study was performed with a semi-automated software, and the clinical feasibility of it had been demonstrated before [7]. FFRCT had shown a higher diagnostic performance compared with CCTA, mainly contributed to an improvement in the specificity and positive predictive value, especially with a substantial 41% reduction in false positive findings [20]. FFRCT also demonstrated superior diagnostic accuracy compared with SPECT or PET in some prospective studies [21, 22].

Several limitations were in current study. First, the major type of MACE was revascularization, while other types were relatively small, which might lead to an impact on the independent predictors. Furthermore, although the retrospective study used PSM to exclude selection bias, the sample size is relatively small, the patients of CCSC grade 2 was more in PCI group and STEMI patients were excluded. This confounds the results. Finally, patients undergoing multi-vessel PCIs and CABG were excluded in current study, thus the findings may be not generalized.

## Conclusion

The results of our study imply that history of unstable angina and FFRCT value ≤ 0.8 were the independent risk factors for MACE, while PCI therapy was the independent protective factor for MACE.

## Data Availability

The datasets generated and/or analysed during the current study are not publicly available due to the fact that it is a preliminary study and the ethics committee does not agree to release the data but are available from the corresponding author on reasonable request.

## Abbreviations

CABG: Coronary artery bypass grafting
CAD: Coronary artery disease
CCSC: Canadian cardiovascular society class
CCTA: Coronary computed tomography angiography
FFR: Fractional flow reserve
FFRCT: Fractional flow reserve derived from computed tomography
MACE: Major adverse cardiac events
MI: Myocardial infarction
PCI: Percutaneous coronary intervention
PSM: Propensity score matching
